# Limited Genetic Connectivity between Gorgonian Morphotypes along a Depth Gradient

**DOI:** 10.1371/journal.pone.0160678

**Published:** 2016-08-04

**Authors:** Federica Costantini, Andrea Gori, Pablo Lopez-González, Lorenzo Bramanti, Sergio Rossi, Josep-Maria Gili, Marco Abbiati

**Affiliations:** 1 Dipartimento di Scienze Biologiche, Geologiche ed Ambientali (BiGeA) & Centro Interdipartimentale di Ricerca per le Scienze Ambientali (CIRSA), University of Bologna, CoNISMa, Via S. Alberto 163, I-48123, Ravenna, Italy; 2 Departament d’Ecología, Facultat de Biologia, Universitat de Barcelona, Av. Diagonal 643, 08028, Barcelona, Spain; 3 Biodiversidad y Ecología de Invertebrados Marinos, Departamento de Zoología, Facultad de Biología, Universidad de Sevilla, Av. Reina Mercedes 6, 41012, Sevilla, Spain; 4 Sorbonne Universités, UPMC Univ Paris 06, CNRS, Laboratoire d'Ecogéochimie des Environnements Benthiques (LECOB), Observatoire Océanologique, 66650, Banyuls sur Mer, France; 5 Institut de Ciència i Tecnologia Ambientals, Universitat Auntònoma de Barcelona, Cerdanyola del Vallés, Spain; 6 Institut de Ciències del Mar–CSIC, Pg. Maritim de la Barceloneta 37–49, 08003, Barcelona, Spain; 7 Consiglio Nazionale delle Ricerche, Istituto di Scienze Marine, ISMAR, Bologna, Italy; University of Padova, ITALY

## Abstract

Gorgonian species show a high morphological variability in relation to the environment in which they live. In coastal areas, parameters such as temperature, light, currents, and food availability vary significantly with depth, potentially affecting morphology of the colonies and the structure of the populations, as well as their connectivity patterns. In tropical seas, the existence of connectivity between shallow and deep populations supported the hypothesis that the deep coral reefs could potentially act as (reproductive) refugia fostering re-colonization of shallow areas after mortality events. Moreover, this hypothesis is not so clear accepted in temperate seas. *Eunicella singularis* is one of the most common gorgonian species in Northwestern Mediterranean Sea, playing an important role as ecosystem engineer by providing biomass and complexity to the coralligenous habitats. It has a wide bathymetric distribution ranging from about 10 m to 100 m. Two depth-related morphotypes have been identified, differing in colony morphology, sclerite size and shape, and occurrence of symbiotic algae, but not in mitochondrial DNA haplotypes. In the present study the genetic structure of *E*. *singularis* populations along a horizontal and bathymetric gradient was assessed using microsatellites and ITS1 sequences. Restricted gene flow was found at 30–40 m depth between the two *Eunicella* morphotypes. Conversely, no genetic structuring has been found among shallow water populations within a spatial scale of ten kilometers. The break in gene flow between shallow and deep populations contributes to explain the morphological variability observed at different depths. Moreover, the limited vertical connectivity hinted that the refugia hypothesis does not apply to *E*. *singularis*. Re-colonization of shallow water populations, occasionally affected by mass mortality events, should then be mainly fueled by larvae from other shallow water populations.

## Introduction

Marine modular organisms exhibit a large morphological variability, with phenotypic plasticity a likely source of this variability (see [1] for a review). In corals and gorgonians variability has led to considerable confusion regarding species boundaries and taxonomy. Genetic investigations can contribute to the recognition of cryptic species boundaries and population identification. Comprehensive studies combining morphological, ecological and genetic data can clarify whether genetic differentiation underlies morphological variation. Moreover, the integrative approach can help to resolve and ⁄or revise taxonomic affinities among closely related species [2]).

Many environmental parameters (e.g. hydrodynamics, salinity, irradiance, trophic resources) can influence organisms’ morphology and drive genetic differentiation [3]. Each parameter exerts its influence independently, but they may act synergistically and their effect is more evident along environmental gradients (e.g. latitude, depth). Indeed, it is widely accepted that the depth gradient, integrating several environmental parameters (e.g. temperature, light, hydrodynamics), may affect population structure, colony morphology, and connectivity patterns of corals and gorgonians [4–7].

Occurrence of high connectivity between shallow and deep populations gave rise to the hypothesis that deep coral reefs can potentially act as (reproductive) refugia [8], fostering the re-colonization of shallow areas after mortality events [9]. Recent studies conflict with this hypothesis, by showing that depth related patterns of genetic structuring vary according to species and sites [10,11]. Prada *et al*. [7] showed that colonies of the tropical gorgonian *Eunicea flexuosa* exposed to high water motion in shallow habitats compared to those living in deeper habitats are taller, have bigger calices, thicker branches and developed on a single plane. Shallow and deep colonies of *E*. *flexuosa* belong to two genetic lineages [7], while within lineages high connectivity was observed among populations across geographic distances of thousands of kilometers. Depth adaptive divergence generates a pattern where neutral genetic divergence is primarily partitioned by habitats, with little geographic structure [12]. Adaptation to local environmental conditions have been also suggested as a driver of genetic structuring across habitats in the tropical corals *Seriatopora hystrix* [10] and *Montastraea cavernosa* [13].

**Fig 1:**
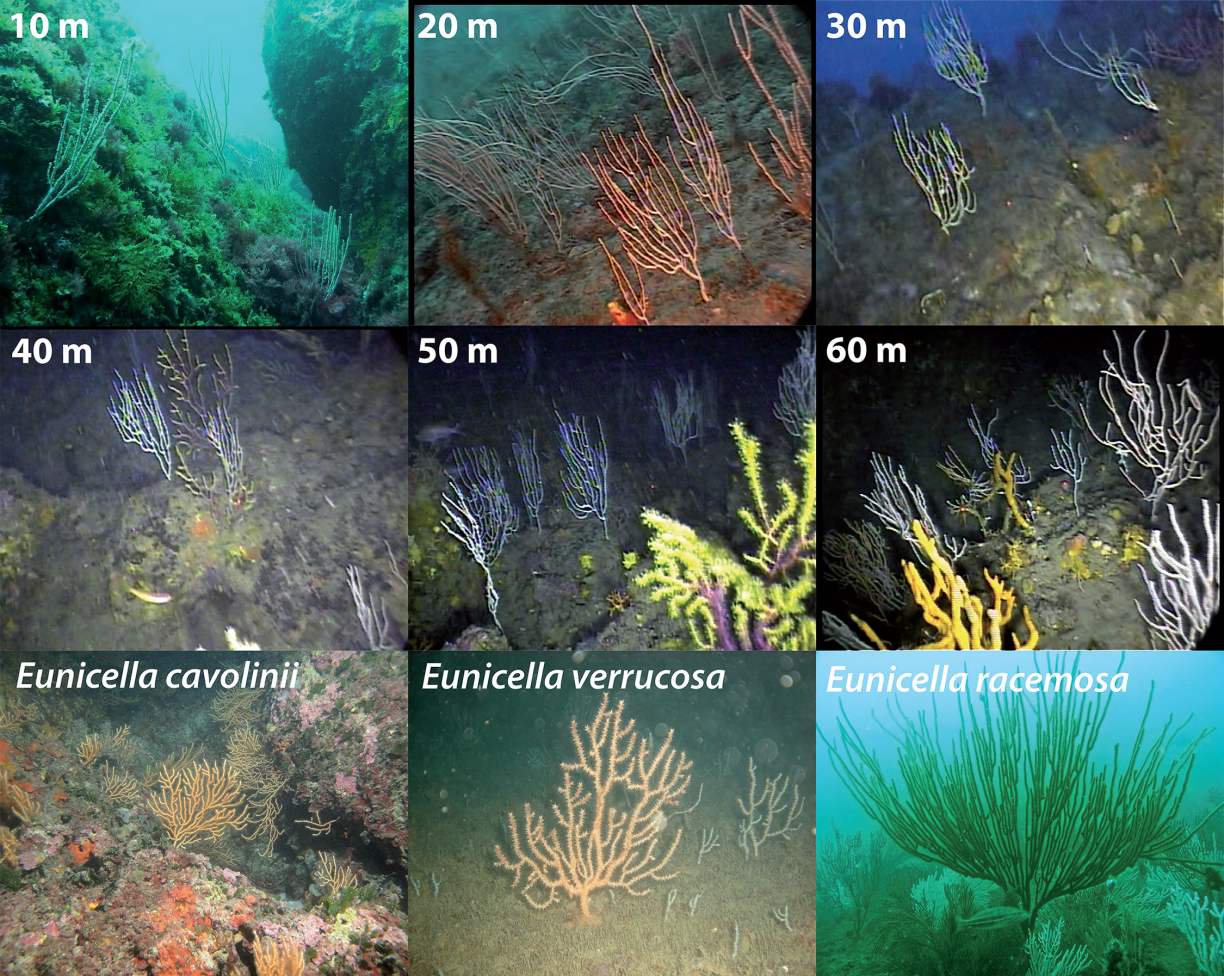
Colonies of *Eunicella singularis* from 10 to 60 meter depth sampled in Cap de Creus; *Eunicella cavolinii* from Elba Island at 18–20 m depth, *E*. *verrucosa* from Tarragona at 13–17 m depth, and *E*. *racemosa* along the Morocco coast. Reprinted from [16], under a CC BY license, with permission from SPRINGER, original copyright 2012

Gorgonian corals of the genus *Eunicella* are among the most representative ecosystem engineers providing biomass and structural complexity in sublittoral communities. Among the 18 recognized *Eunicella* species, 6 species are present in the Mediterranean Sea: *E*. *singularis*, *E*. *cavolinii*. *E*. *verrucosa*, *E*. *filiformis*, *E*. *gazella* and *E*. *labiata*. The distribution of the last three species is limited to the Strait of Gibraltar and to the Alboran Sea, and they are considered mainly Atlantic. *E*. *cavolinii* and *E*. *singularis* occur both in the western and eastern Mediterranean Sea, being the later also present in few locations along the Atlantic coast near the Strait of Gibraltar. *E*. *singularis* is the only species showing a high morphological variability along a depth gradient from 10 to 70 m in the western Mediterranean Sea [14,15] (Fig 1; [16]).

A shallow and a deep morphotype of *E*. *singularis* have been identified (see [17], [16], [18] for a detailed description of the geographical and bathymetric distribution, and the variability of the two morphotypes). The two morphotypes significantly differ in colony/sclerite shape, as well as in trophic ecology [19] and for the presence/absence of symbiotic algae [16]. Théodor [17] associated the deep morphotype to the aposymbiotic (facultative symbiotic) form of *E*. *singularis*, raising a question about its taxonomic status. Just as for the other gorgonians [20–22], mitochondrial DNA in *Eunicella* species has a very low mutation rate and is not a useful marker to discriminate between the two *E*. *singularis* morphotypes as well as among the different Mediterranean species of *Eunicella* [16,21]. To date, a detailed phylogenetic analysis within the *Eunicella* genus is missing, and the taxonomic status of the two *E*. *singularis* morphotypes has not been clarified yet [16]. The objective of the present study is to test whether along the depth gradient morphological differences observed in *E*. *singularis* match with genetic differentiation. To this aim a population genetic approach using microsatellites and ITS1 sequence polymorphism was carried out. Moreover, divergence pattern within the genus were investigated by addressing the phylogenetic relationships among the *Eunicella* species. To assess the level of vertical connectivity, and the variation of the genetic parameters with depth samples of *E*. *singularis* were collected every 10 m from 10 to 60 m depth in the only site where it occurs on a vertical cliff (Cap de Creus, Northwestern Mediterranean Sea). Eight further samples were collected in the same area along about 15 km of coast at 15–20 m depth to assess the horizontal pattern of genetic connectivity, and to be able to compare the 2 patterns. Finally, the phylogenetic relationships between *E*. *singularis*, the Mediterranean species *E*. *cavolinii* and *E*. *verrucosa*, and the Atlantic *E*. *racemosa*, were analyzed to scale their genetic distances.

## Materials and Methods

### Ethic statement

Permissions to collect the gorgonian samples were requested to the local authorities at each site: the Parc Natural de Cap de Creus for the Cap de Creus MPA; the Societat d'Exploracions Submarines de Tarragona for Tarragona; the Parco Nazionale dell’Arcipelago Toscano for Elba Island, and the Université Ibn Zohr Agadir-Maroc for Taghazout. The field studies did not involve endangered or protected species.

### Study area and sampling design

**Fig 2:**
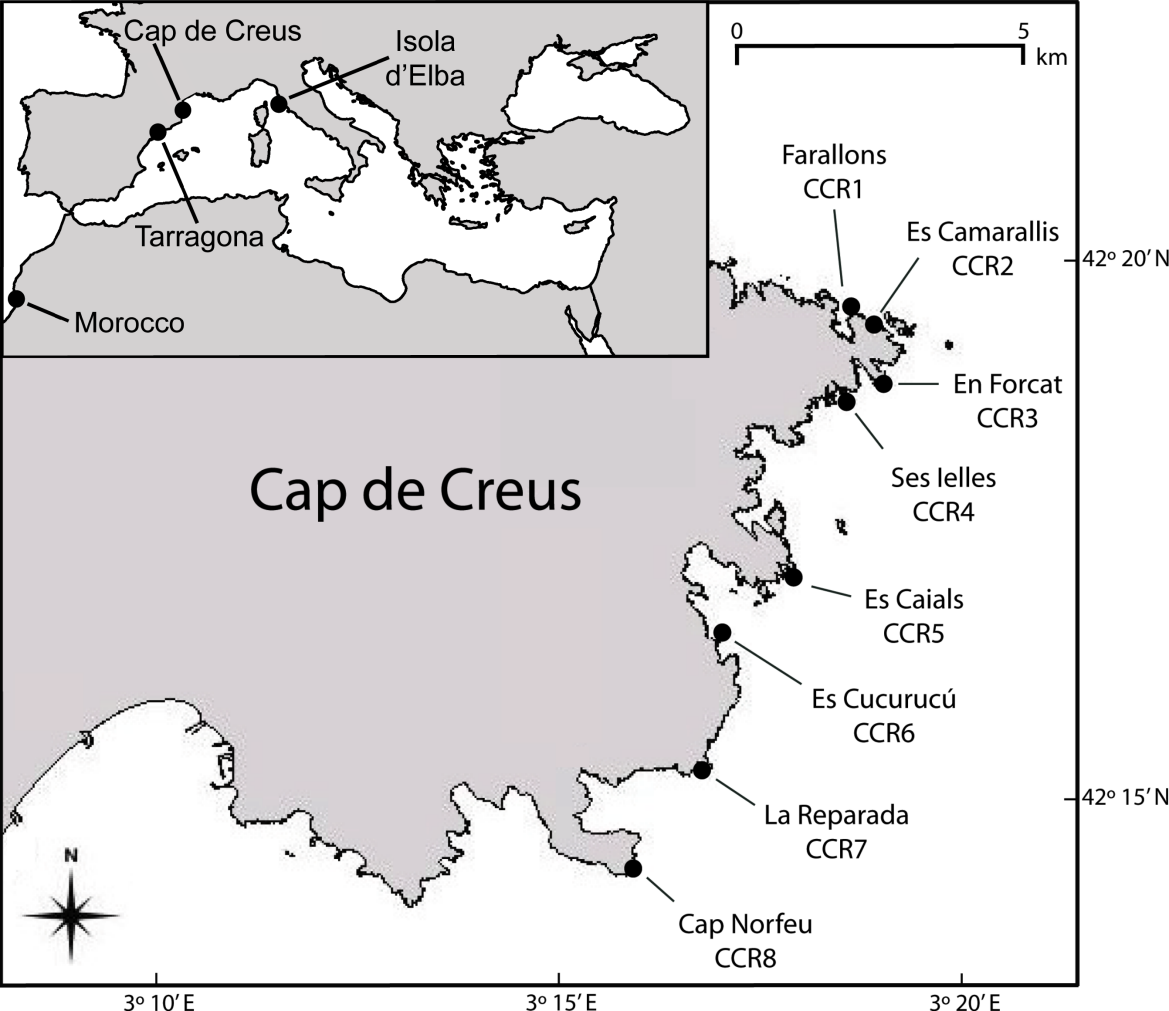
Map of the sampling areas where the *Eunicella* species where collected, and position of the 8 locations where *Eunicella singularis* colonies were sampled along the Cap de Creus coast. In Els Forcats (CCR3) colonies were collected at 6 depths along a sloping rocky bottom from 10 to 60 m depth (named CCR3_10 to CCR3_60). Reprinted from [16], under a CC BY license, with permission from SPRINGER, original copyright 2012.

The study was planned at Cap de Creus (42°18’44’N; 003°19’05”E), where *Eunicella singularis* is the most common and abundant gorgonian species occurring at high densities in sublittoral rocky bottoms from 10 to 70 m depth [18]. Cap de Creus is characterised by vertical cliffs. However, after a careful search, only in one accessible site (Els Forcats; CCR3) *E*. *singularis* occurred along a vertical transect from 10 to 60 m depth. In this site 16 branch fragments were collected from different colonies every 10 m depth (Fig 2, Table 1). Furthermore, in eight locations along 15 km of Cap de Creus coast, branch fragments were collected by SCUBA diving from about 30 colonies of *E*. *singularis* at 18–20 m depth (Fig 2; Table 1). Finally, to analyse the genetic diversity and phylogenetic relationship in the *Eunicella* genus, a small branch fragment was sampled from 5 different colonies of: *E*. *cavolinii* at Elba Island (42°49’18”N; 010°09’52”E, Central Mediterranean) at 18–20 m depth, *E*. *verrucosa* at Tarragona (41°06’07”N; 001°15’12”E, Northwestern Mediterranean) at 13–17 m depth, and *E*. *racemosa* along the Morocco coast (30°32’51”N; 9°43’59”W, Taghazout, East Atlantic Ocean) at 15 m depth. All the collected material was preserved in 80–100% ethanol and stored at 4°C pending analysis.

**Table 1:** Geographical coordinates of the sampling locations and depth range at which *E*. *singularis* colonies were collected. N: numbers of analysed colonies.

Location	Code	Lat.	Long.	Depth (m)	N
Farallons	CCR1	423.247	33.111	18–21	30
Es Camarallis	CCR2	423.230	33.186	18	30
Els Forcats	CCR3_10	423.144	33.186	10	16
Els Forcats	CCR3_20	“	“	20	16
Els Forcats	CCR3_30	“	“	30	16
Els Forcats	CCR3_40	“	“	40	16
Els Forcats	CCR3_50	“	“	50	16
Els Forcats	CC3_60	“	“	60	16
Ses Ielles	CCR4	423.119	32.977	17–20	30
Es Caials	CCR5	422.841	32.977	15–17	30
Es Cucurucú	CCR6	422.733	32.844	15–17	30
La Reparada	CCR7	422.511	32.808	17	25
Cap Norfeu	CCR8	422.391	32.675	20	30

### Microsatellite genotyping and ITS1 amplification

Total genomic DNA was extracted from three to five polyps per colony fragments following the cetyl trimethyl ammonium bromide (CTAB) procedure [23]. After DNA extraction, all the individuals were genotyped using 6 microsatellite loci developed by [24]: C21, C30, C40 and [25]: EVER1, EVER3, EVER9 following their protocols. Genotyping of individuals was carried out on an ABI 310 Genetic Analyser (Applied Biosystems), using forward primers labelled with FAM, HEX, TAMRA, ROX (Sigma) and LIZ HD500 (Applied Biosystems) as internal size standard through MACROGEN INC. Service. Allele sizing was determined using PEAK SCANNER v.1.0 software from Applied Biosystems, Inc.

For ITS1 sequence analysis, only individuals belonging CCR1, CCR2, CCR3 (10–60) and CCR4 were amplified. Polymerase chain reaction (PCR) amplifications of the ITS1 region were carried out using the primers ITS1-new-F and ITS1-new-R [26]. Each 25 μL PCR reaction contained approximately 20 ng DNA, 1× PCR buffer (Promega), 2 mm MgCl_2_, 0.5 μm of each primer, 0.4 mm dNTPs and 1 U of Taq polymerase (Promega). Amplifications were performed on a GeneAMP PCR System 2700 (Applied Biosystems) as follows: an initial denaturation at 95°C for 3 min, 30 cycles including 95°C for 30 s, 57°C for 30 s and 72°C for 60 s. A final extension at 72°C for 7 min was added. PCR products were sent to Macrogen (South Korea) for purification and sequencing since some sequences contained several heterozygous sites, ITS1 sequence types were estimated using PHASE 2.1 [27] on DNASP v. 5 [28], which implements a coalescent-based Bayesian method to infer them. Both alleles of all individuals were included in the alignment (286 reference individuals). Alignment was made manually using the biological sequences alignment editor BIOEDIT v. 7.2.5.

### Genetic variability: microsatellite and ITS

Linkage disequilibrium analysis for pairs of loci was based on the likelihood ratio test with the EM algorithm [29] through 10,000 permutation procedures (number of initial conditions for EM, 100) using ARLEQUIN v. 3.5 [30]. Individuals sharing the same multilocus genotype (MLG) were checked using GENALEX v. 6.1 [31]. Identical MLGs can be the result of two different genotypes originated by two distinct sexual reproduction events but sharing the same alleles for all genotyped loci. The unbiased probability of identity (P_ID_) [32] was computed to test this possibility that two sampled individuals share identical MLG by chance through sexual reproduction and not to clonal reproduction or sampling errors due to the collections of the same colony. Genetic diversity within site for each locus and over all loci was estimated as observed (H_O_) and expected (H_S_) heterozygosity using the GENETIX software package v. 4.05 [33]. Allelic richness (Ar) and private allelic richness (Ap) were calculated with a rarefaction procedure using the HP-Rare software [34]. Single- and multilocus F_IS_ values were estimated using Weir & Cockerham’s F [35], and significant departures from the Hardy–Weinberg equilibrium were tested using “Fisher’s exact test” in GENEPOP v. 3.4 [36] as implemented for online use (http://genepop.curtin.edu.au/), with the level of significance determinate by a Markov-chain randomization. The presence of null alleles was estimated using the expectation maximization (EM) algorithm of [29] in FREENA [37]. BOTTLENECK v. 1.2.02 [38] was used to test for recent demographic changes. Significant differences between He and Heq–heterozygosity under mutation-drift equilibrium calculated using two-phase model (TPM) with 95% of the stepwise mutation model (SMM) and variance among multiple steps equal to 12 [38] were tested using Wilcoxon’s signed rank-test. A one-way full-factorial permutation multivariate analysis of variance (PERMANOVA, [39]) was used to test the null hypothesis of no difference in genetic diversity indexes (observed heterozygosity, allelic richness and private allelic richness) between depths using the 6 loci as replicates. Data were normalized (after transformation of private allelic richness as logarithm plus one), and the Euclidean distance calculated between each pair of samples. A total of 9,999 permutations were used under a reduced model. The analysis was followed by pairwise comparisons to test for differences between depths. Sequence genetic diversity within samples was estimated as number of haplotypes (h), haplotype and nucleotide diversity (Hd and π, respectively). All these parameters were calculated through DNASP. To display evolutionary relationships between sequence types (ST), ITS1 sequences were represented in a haplotype network calculated by Median Joining with the software Network v. 4.6.1.1 [40].

### Population genetic structure

For both molecular markers, the genetic divergence among populations was estimated using Weir & Cockerham [35] *F*_*ST*_ estimator in ARLEQUIN. Genotypic differentiation among populations was tested with an exact test implemented in GENEPOP (Markov chain parameters: 1,000 dememorizations, followed by 1,000 batches of 1,000 iterations per batch). Since the presence of null alleles, genetic divergence among samples at microsatellite loci was estimated in FREENA using the F_ST_ estimates of Weir [41] and following the so-called ENA method described in [37] which provides unbiased *F*_*ST*_ estimates, computed excluding null alleles. To evaluate the number of clusters (K) in the dataset without prior information regarding the geographic distribution of the samples, a Bayesian method implemented in STRUCTURE v. 2.3.4 [42,43] was used under the admixture model and choosing the assumption of correlated allele frequencies and the option of recessive alleles to cope with null alleles, as suggested by Falush [43]. Mean and variance of log likelihoods of the number of clusters for K = 1 to K = 10 were inferred from multilocus genotypes. Ten runs were performed for each K with 500,000 iterations and a burn-in period of 50,000. In order to identify the number of clusters that best fit the data, the resulting output files were then analysed using the Evanno method in STRUCTURE HARVESTER v. 0.6.94, as implemented for online use (http://taylor0.biology.ucla.edu/structureHarvester/) [44]. Moreover, the STRUCTURE results were summarized using CLUMPAK [45] to obtain the probability of each individual to belong to each cluster. A discriminating analysis of principal components (DAPC) as implemented in the ADEGENET software v. 1.3 [46] was performed. This technique extracts information from genetic datasets (multivariate in nature) by first performing a principal component analysis (PCA) on pre-defined groups or populations, and then using the PCA factors as variables for a discriminating analysis (DA), which seeks to maximize the intergroup component of variation. The optimal number of clusters (populations) was predicted using the k-means clustering algorithm, find clusters, retaining all principal components. In all analyses, 20 principal components of PCA were retained as input to DA, which accounted for approximately 96% of the total genetic variability. Significance levels for multiple comparisons of loci across samples were adjusted using a false discovery rate (FDR) correction for multiple tests [47]. Hierarchical analysis of molecular variance (AMOVA) was conducted in ARLEQUIN in order to quantify genetic variation between two groups depending on the depth: shallow populations and deep populations (CCR3_40, CCR3_50 and CCR_60).

Significance of F-statistics was achieved using 99,999 permutations. Individual assignment tests were performed using the program GENECLASS v. 2 [48]. This Bayesian procedure computes the likelihood of a genotype in a given population assuming an equal prior probability density to the allelic frequencies of each locus in each population. Bayesian method [49] and the re-sampling algorithm of Cornuet *et al*. [50] set at 1,000 individuals were used. An individual was excluded from a given candidate population if its probability of belonging to a particular population was lower than 5%. To evaluate the extent of contemporary (ecological time-scale) dispersal of *E*. *singularis* among sites, the numbers of first-generation migrant were also calculated through the program GENECLASS v. 2.

### Phylogenetic analyses

To perform a preliminary phylogeny of the genus *Eunicella*, a dataset with all the individual ITS1 sequences from the different species here examined was created. Distance matrixes of sequence divergence among the species were calculated as p distance (Dp) in MEGA v. 5.05.

The best-fit substitution model for the dataset was calculated using JMODELTEST v. 1.1 [51] Mac software, considering 88 substitution models by hLRT calculator with 4-gamma category. Phylogenetic relationship among species were carried out using a Bayesian approached implemented in MrBayes v. 3.1.2 software using the Hasegawa, Kishino and Yano substitution model. The analysis was carried out for 2,000,000 generations, sampling every 1,000 generations. The first 500 of sampled generations were discarded as the burn in. Phylogenetic tree were visualized as posterior probability using FIGTREE v. 1.4.0. ITS1 sequences of *Corallium rubrum* (GenBank accession number: FJ87608282 and FJ87608283) were used as outgroup.

## Results

### Genetic variability: microsatellite and ITS

301 individuals were genotyped for the 6 loci. Tests of linkage disequilibrium between loci within populations indicated no significant association of alleles (all *p* > 0.05), confirming that all loci are independent markers. Six shared multilocus genotypes (MLGs) between colonies were found. Five MLGs were encountered twice in CCR3_40, CCR3_50 and CCR3_60. Within CCR3_50 one MLG was encountered five times. In CCR3_50 the probability that each of these genotypes was produced by chance alone was high (P_ID_ = 4.6 e-02).

The allelic richness (Ar) based on a minimum sample size of 22 genes, ranged from 2.24 (in CCR3_50) to 4.25 (in CCR7), while the private allelic richness (Ap) ranged from 0 (in CCR3_20 and CCR3_30) to 0.46 (in CCR7). Expected heterozygosity (H_E_) and observed heterozygosity (H_O_) were lower in CCR3_50 (H_E_ = 0.25, H_O_ = 0.28) and higher in CCR4 (H_E_ = 0.58, H_O_ = 0.57). Three (CCR2, CC3_10 and CCR3_30) out of 13 samples showed significant deviations from HW equilibrium with values of F_IS_ positive and higher than 0.173 (Table 2).

**Table 2:** Genetic diversity of *Eunicella singularis* at six microsatellite loci; H_O_: observed heterozygosity, H_E_: unbiased expected heterozygosity, Ar: allelic richness, Ap: private allelic richness, F_IS_: Weir and Cockerham’s [35] estimate fixation index with significant values in bold (0.05 threshold after FDR correction).

Site	H_O_	H_E_	Ar	Ap	F_IS_
CCR1	0.50	0.57	3.68	0.18	0.115
CCR2	0.47	0.57	3.85	0.02	**0.173**
CCR3_10	0.44	0.55	3.43	0.06	**0.205**
CCR3_20	0.52	0.52	3.21	0.00	0.07
CCR3_30	0.47	0.59	3.42	0.00	**0.203**
CCR3_40	0.54	0.53	3.39	0.15	**-**0.028
CCR3_50	0.28	0.25	2.24	0.05	**-**0.098
CCR3_60	0.49	0.51	3.46	0.13	0.045
CCR4	0.57	0.58	3.70	0.07	0.011
CCR5	0.55	0.58	4.29	0.21	0.053
CCR6	0.52	0.54	3.62	0.05	0.043
CCR7	0.52	0.57	4.25	0.46	0.096
CCR8	0.53	0.58	3.98	0.15	0.083

Estimated null allele frequencies ranged between loci from 0.022 ± 0.035 in EVER9 to 0.0748 ± 0.087 in C21. The Wilcoxon’s signed rank-test, performed in BOTTLENECK showed no demographic population changes in the recent past for all samples (data not shown). The one-way PERMANOVA showed that the total genetic variability differed by depth (MS = 6.68; F = 2.65; df = 5; *p* = 0.005). The pairwise test located a threshold of genetic variability at 40 m depth (10–50 t = 2.42, *p* = 0.0097; 20–50 t = 3.86, *p* = 0.0001; 30–50 t = 2.88, *p* = 0.0054).

Samples collected in 3 shallow sites (CCR1, CCR2, CCR4) and along the vertical gradient in CCR3 (CCR3_10 to CCR3_60) were analyzed using ITS1 sequence polymorphism. In total, 286 ITS1 sequences corresponding to 143 individuals were analyzed. The length of the amplified ITS1 fragment was 200 bp with 9 variable sites at the positions 14, 21, 114, 119, 158, 159 and 173, corresponding to 9 sequence types (ST1 to ST9; Table 3).

**Table 3:** Sequence types frequencies, including heterozygous individuals in each population of *E*. *singularis*. H: number of haplotypes (in the sense of sequence type); Hd: haplotype diversity; πd: nucleotide diversity.

	CCR1	CCR2	CCR£_10	CCR3_20	CCR3_30	CCR3_40	CCR3_50	CCR3_60	CCR4
ST1	26	22	31	31	31	14	32	16	26
ST2	4								6
ST3		7							
ST4		3							
ST5			1		1				
ST6				1					
ST7						18		10	
ST8								4	
ST9								2	
H	2	3	2	2	2	2	1	4	2
Hd	0.024±0.092	0.486±0.085	0.063±0.058	0.063±0.058	0.006±0.058	0.508±0.031	0	0.653±0.057	0.315±0.087
πd	0.00120	0.00398	0.00124	0.00063	0.00031	0.00254	0	0.00415	0.00157

**Fig 3:**
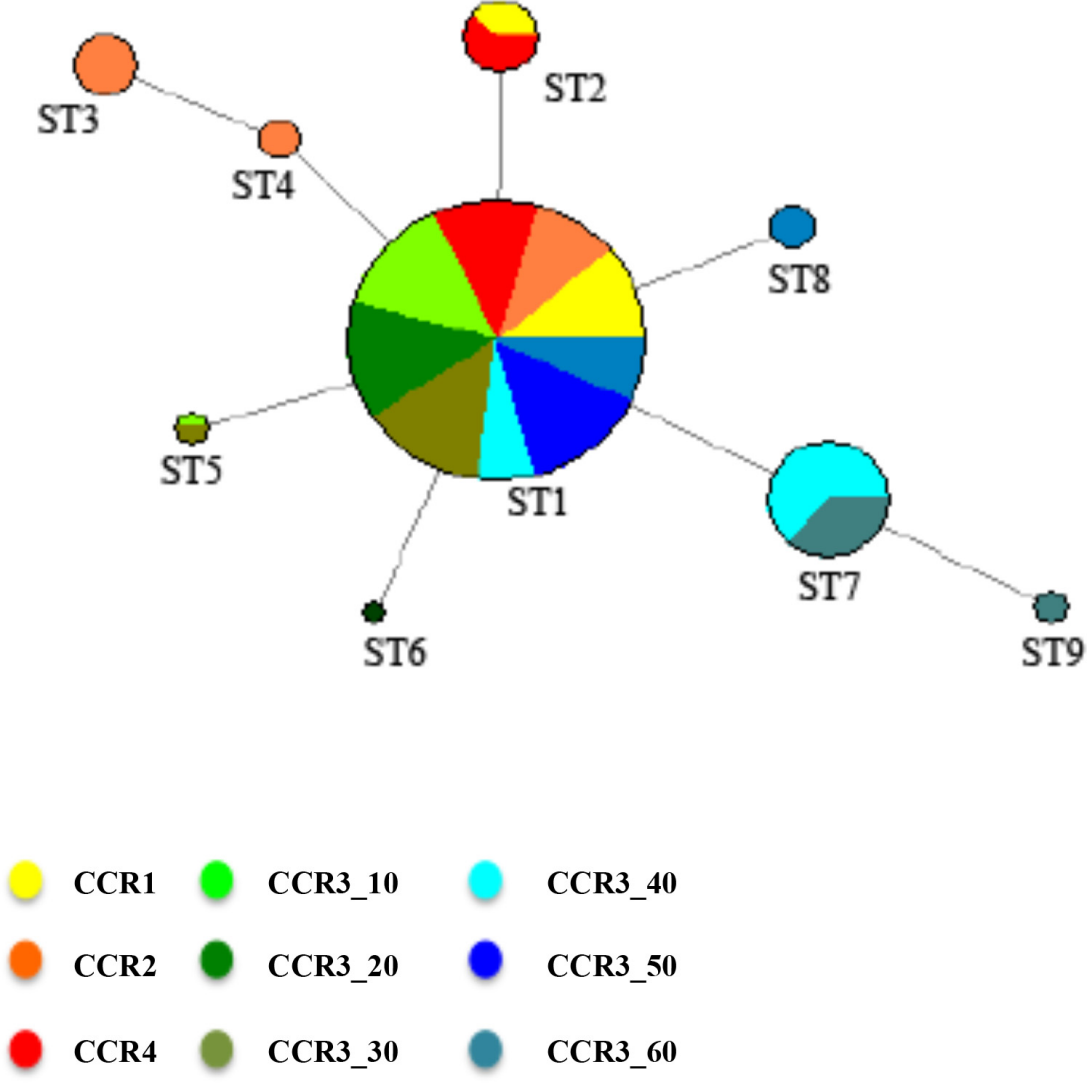
Median-joining network depicting relatedness and geographic distribution of ITS1 sequence type (ST) of *Eunicella singularis*. Circle size is proportional to the number of colonies with the corresponding sequence type. ST means “sequence type” to refer to every distinct type of ITS-1 sequence detected.

The more frequent sequence type was ST1 occurring in all populations, while the second more frequent sequence type was ST7 occurring in CCR3_40 and CCR3_60 with 9 and 5 individuals, respectively (Table 3; Fig 3). Haplotype diversity ranged from 0 in CCR3_50 to 0.653 in CCR3_60 (Table 3). No significant differences between CCR3_10, CCR3_20, CCR3_30 *vs*. CCR3_40, CCR3_50, CCR3_60 were observed in the haplotype diversity (Student’s t-test, *p* = 0.07; data not shown). The sequence type network showed a star-shape pattern with a high frequency dominant haplotype with at the center. Derivatives with lower frequencies were connected to the dominant haplotypes by only one mutation step (Fig 3).

### Population genetic structuring

**Fig 4:**
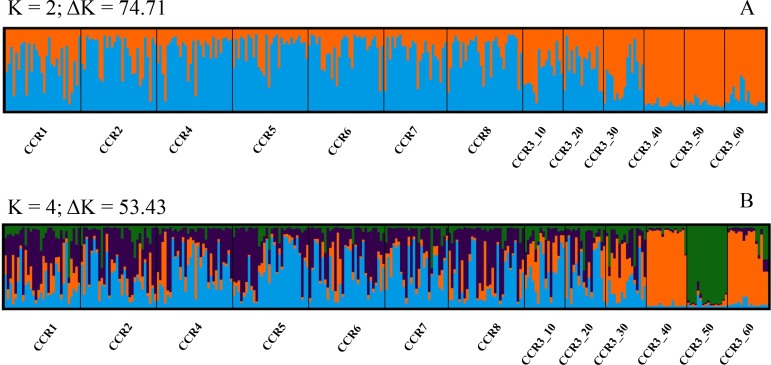
Population structure as inferred by the STRUCTURE analysis for K = 2 (A) and K = 4 (B) clusters in *E*. *singularis*. Individuals are represented by vertical bars; the colours correspond to different genetic clusters, and the colours proportions of the individuals indicate their membership (from 0 to 1) in the corresponding cluster. Each graph corresponds to the combination of ten different runs obtained for each K value. Population codes are indicated in Table 1.

**Fig 5:**
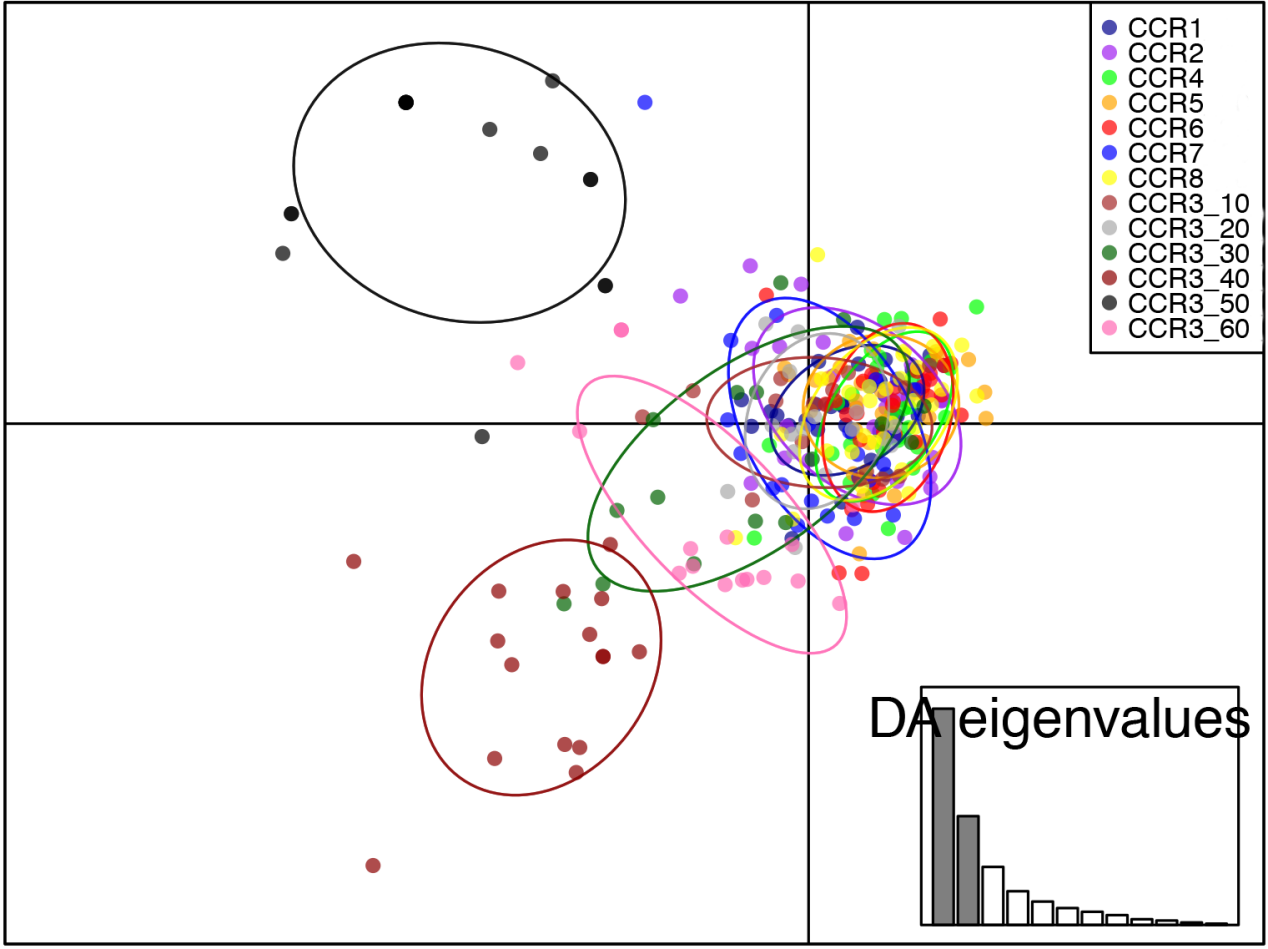
Subdivision of the *Eunicella singularis* colonies according to the DAPC method. Colonies from different sites are indicated with different colours, dots represent individual colonies. A barplot of eigenvalues for the discriminant analysis is displayed in inset. Eigenvalues show the amount of genetic information contained in each successive principal component with x and y-axes constituting the first two principal components, respectively.

Genotypic differentiation between samples and *F*_*ST*_ estimates based on the ENA method gave similar results to those obtained when presence of null alleles was not taken into account. Pairwise *F*_*ST*_ values ranged from -0.002 (CCR2 *vs*. CCR5) to 0.482 (CCR3_50 *vs*. CCR3_60). After FDR correction, all the pairwise comparison with CCR3_40, CCR3_50 and CCR3_60 were significant (Table 4). During the first round of STRUCTURE, two genetic clustering were identified as more plausible, K = 2 (∆K = 74.71) (Fig 4A). When K = 2 is considered, the first cluster includes CCR3_40, CCR3_50 and CCR3_60, while the other cluster includes all the remaining samples. When K = 4 is considered, STRUCTURE outline a new genetic pool characterized corresponding to CCR3_50 (Fig 4B). The DAPC confirmed the genetic differentiation observed using Structure. In the data transformation step for PCA analysis, 20 principal components (PCs) were retained, accounting for approximately the 95% of the total genetic variability. The eigenvalues of the DAPC indicated that the first two components explained most of the variation. Along the first axis CCR3_40, CCR3_50 and CCR3_60 were separated from the others samples; the second axis contrasts CCR3_50 with CCR3_40 and CCR3_60 (Fig 5). In fact, 93% of individuals were reassigned to their original clusters CCR3_50 and CCR3_40, while 75% of the individuals were reassigned to CCR_60. The values of the proportions of successful reassignment were below 30% in all the shallow populations suggesting admixture among them.

**Table 4:** Pairwise multilocus estimates of microsatellite *F*_*ST*_ between all *Eunicella singularis* populations. Values in bold indicate significance after correction for multiple testing.

	CCR1	CCR2	CCR3_10	CCR3_20	CCR3_30	CCR3_40	CCR3_50	CCR3_60	CCR4	CCR5	CCR6	CCR7
**CCR2**	0.027											
**CCR3_10**	0.059	0.052										
**CCR3_20**	0.055	0.036	0.028									
**CCR3_30**	0.051	0.056	0.042	0.035								
**CCR3_40**	**0.274**	**0.298**	**0.280**	**0.273**	**0.221**							
**CCR3_50**	**0.316**	**0.359**	**0.414**	**0.382**	**0.334**	**0.444**						
**CCR3_60**	**0.203**	**0.239**	**0.196**	**0.253**	**0.200**	**0.202**	**0.482**					
**CCR4**	0.031	0.011	0.046	0.040	0.063	**0.272**	**0.356**	**0.248**				
**CCR5**	0.046	-0.002	**0.091**	0.044	**0.083**	**0.318**	**0.366**	**0.267**	0.025			
**CCR6**	0.025	0.002	**0.066**	**0.064**	**0.079**	**0.323**	**0.392**	**0.275**	0.010	0.015		
**CCR7**	**0.067**	0.014	**0.092**	0.054	**0.091**	**0.281**	**0.381**	**0.265**	0.015	0.006	0.018	
**CCR8**	0.031	0.007	**0.081**	0.053	0.047	**0.283**	**0.351**	**0.227**	0.015	0.019	0.022	0.028

The AMOVA supported the clustering structure showing a significant difference between shallow and deep groups: 15.39%, *p* < 0.05 (Table 5).

**Table 5:** Analysis of molecular variance (AMOVA) among samples of *E*. *singularis* using microsatellite and ITS-1 data sets. For the ITS-1 dataset only CCR1, CCR2, CCR3 and CCR4 were analysed. *E*. *singularis* samples were grouped according their depth (two groups: shallow populations and CCR3_40, CCR3_50, CCR3_60), *P < 0.05, **P < 0.001

	Microsatellites			ITS1		
Source of variation	df	Variance components	%	df	Variance components	%
Among groups	1	0.313	15.39**	1	0.041	16.66**
Among populations within groups	11	0.148	7.25**	7	0.043	17.43**
Within populations	589	1.577	77.36**	277	0.163	65.91*

Of the 301 individuals included in the assignment analysis, 38.2% were assigned to their actual sampling location. In particular, all individuals from CCR3_40, CCR3_50 and CCR3_60 were assigned to their population of origin. The analysis of first generation migrants, based on the Bayesian computation of Rannala et al. [49] implemented in GENECLASS v. 2, classified 6 colonies as originating from a site different to the one they were collected at. None of the 6 colonies were identified as immigrants coming from deeper populations (data not shown).

For the ITS1 sequence data set, *F*_*ST*_ ranged from 0 (CCR3_10 *vs*. CCR3_20; CCR3_20 *vs*. CCR3_30 and CCR3_50 *vs*. CCR3_10, CCR3_20, CCR3_30) to 0.548 (CCR3_40 *vs*. CCR3_50), and 15 out of the 36 pairwise *F*_*ST*_ estimates were not statistically significant after False Discovery Rate correction. As for the microsatellites, also the AMOVA performed using the ITS1 dataset supported the clustering structure (differences among groups: 16.66%, *p* < 0.05) (Table 5).

### Phylogenetic analyses of the Mediterranean *Eunicella* species based on ITS1

**Fig 6:**
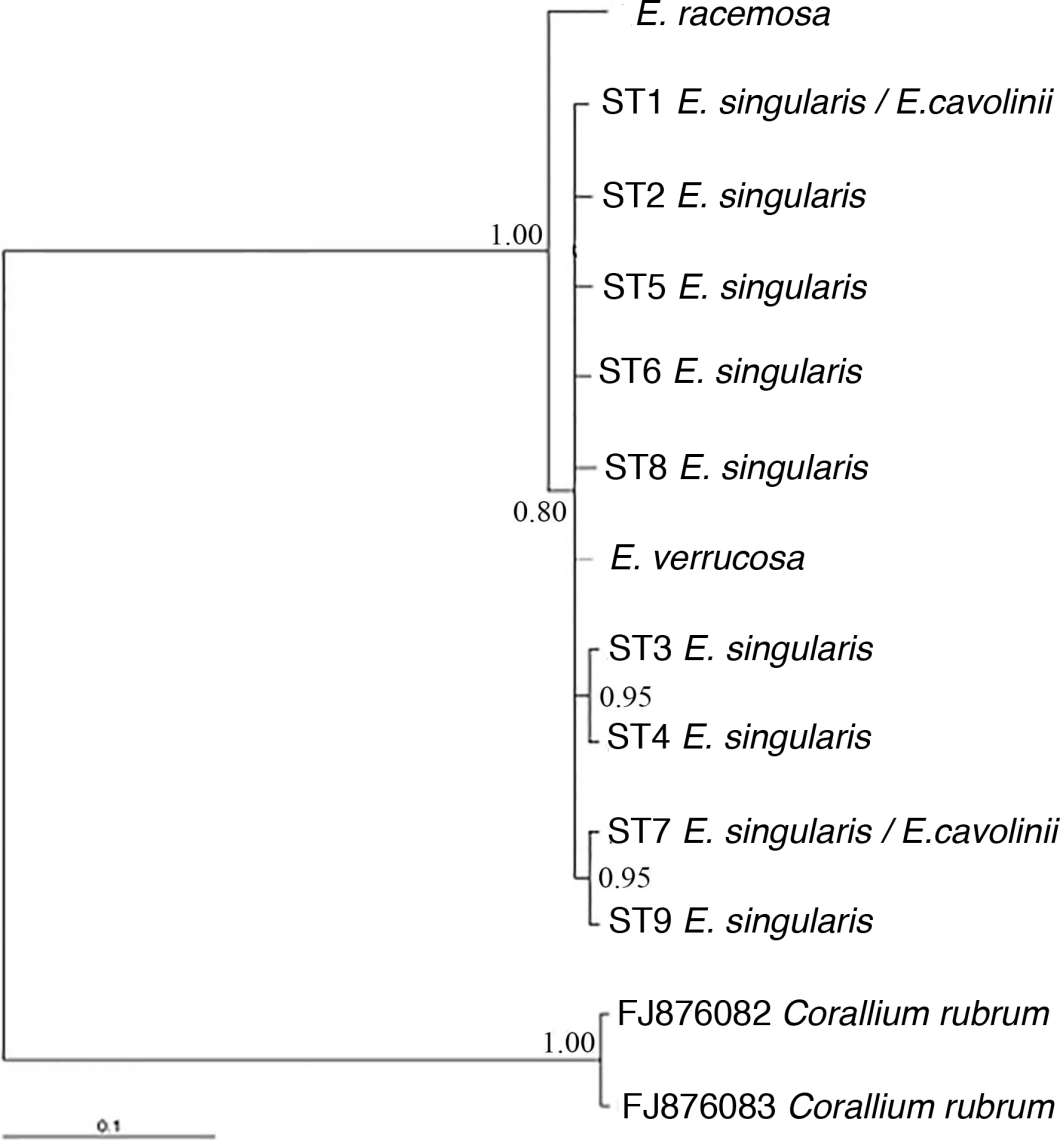
Bayesian inference tree. ITS1 Bayesian inference tree illustrating the phylogenetic relationships among *Eunicella* species. Numbers near nodes are posterior probability (PP) values. ST mean ‘sequence type’ to refer to every distinct type of ITS-1 sequence detected, as proposed by [52].

All the sequences types found in *E*. *singularis* were aligned with the sequences of *E*. *cavolinii*, *E*. *verrucosa* and *E*. *racemosa*. The Atlantic species *E*. *racemosa* differed from the others for the presence of an insertion of 5 bp at the position 22–26. All the 5 colonies of *E*. *verrucosa* and *E*. *racemosa* showed identical sequences. Three colonies of *E*. *cavolinii* present the same sequence of *E*. *singularis* ST1 and the PHASE analysis implemented in DNAsp showed that one colony of *E*. *cavolinii* presented 2 different sequence types: ST1 and ST7 (GenBank accession numbers KX002245-KX002255). The p-distance matrix showed that the greatest divergence among sequences was observed between *E*. *racemosa* and all the other species with values ranging from 4.52% to 5.02% (S1 Table). Moreover, values of the mean genetic distance among all the populations of *E*. *singularis* (Dp = 0.0015 ± 0.0014; ranging from 0.08% to 0.33%) were close to values of the mean genetic distance between *E*. *singularis* and *E*. *cavolinii* (Dp *=* 0.0015 ± 0.0013; ranging from 0.5% to 0.45%). On the other hand, mean p-distance values among shallow (CCR3_10, CCR3_20 and CCR3_30) and deep sites (CCR3_40, CCR3_50 and CCR3_60) were 0.000 and 0.243 ± 0.0002, respectively. Mean difference between shallow and deep sites was 0.0016 ± 0.0012 (S1 Table). However, p-distance data for deep sites are affected by the lack of differentiation between CCR3_50 and all CCR3 shallow sites (CCR3_10, CCR3_20 and CCR3_30) as well as CCR1 and CCR4. The site CCR3_50 only differs from CCR2, CCR3_40 and CCR3_60. ITS1 Bayesian inference tree showed that *Eunicella racemosa* is genetically separated from the others *Eunicella* species (PP = 1.00). Moreover, *E*. *verrucosa* and *E*. *cavolinii* clustered together with the *E*. *singularis* sequence types (Fig 6). Only a couple of grouping among unresolved *E*. *cavolinii* sequence types could be detected (each PP = 0.95), the first one clustering sequence types ST3 and ST4 (both only found in the shallow water site CCR2) and the second one composed by the sequence types ST7 and ST9 (the first one found in CCR3_40 and CCR3_60, while the second is also found in CCR3_60) (see Fig 5 and Fig 6).

## Discussion

The present study reveals that shallow and deep morphotypes of *Eunicella singularis* in Cap de Creus [16,17] correspond to two genetically isolated populations with a boundary located across the 40 m depth. Conversely, shallow water populations do not show genetic structure at a spatial scale of about fifteen kilometers.

Observed phylogenetic patterns within the genus *Eunicella* are more complex than expected, and current morphological and molecular taxonomy cannot clearly discriminate the three 3 common Mediterranean species. Further studies using new polymorphic and variable markers are needed to shed light on the evolutionary history among *Eunicella* lineages.

### Genetic variability and connectivity along the depth gradient

Significant differences in the genetic variability were observed between shallow and deep populations. This variability was mainly explained by the presence of high values of private alleles observed in the populations below 40 m depth. These results are in contrast with the decreasing genetic variability along depth gradient observed in *Corallium rubrum* populations in Cap de Creus [6]. In *C*. *rubrum* decreasing genetic variability has ascribed to the present of recent bottleneck, probably as a consequence of the past overexploitation of deeper populations. In *E*. *singularis* no recent bottlenecks were observed in any population. In fact, in Cap de Creus, rocky bottoms below 40 m depth are characterized by high density of medium-sized and sexually mature colonies [18,53], suggesting a high effective population size and long population persistence. Pairwise *F*_*ST*_ estimates using microsatellite loci, and AMOVA on ITS1 sequences revealed a high degree of genetic differentiation between populations above and below 40 m depth. Structure analysis suggested the presence of a barrier to gene flow across 40 m. Among the deepest populations, CCR3_50 seems the most divergent with a high rate of self-recruitment, suggesting that a nonlinear increase of genetic distance with depth. DAPC and structure analysis with K = 4 also support this hypothesis, showing a higher similarity between colonies at 40 and 60 m depth compared to colonies at 50 m depth. Pey *et al*. [54] in *E*. *singularis* and Pivotto *et al*. [55] in *E*. *cavolinii* did not found any genetic differentiation comparing colonies from 10–15 m to 35–40 m depth, confirming absence of barrier to gene flow in populations above 40 m depth. While *C*. *rubrum* colonies collected along a 20–70 m depth gradient revealed a threshold in connectivity at about 40 m depth [6], suggesting that environmental features associated with depth have an important role in determining patterns of genetic structuring in Mediterranean coastal gorgonians. The present study provides genetic data supporting the differentiation of *E singularis* in two morphotypes (shallow and deep) differing in color, colony shape and sclerites features described by Théodor [17] and Gori *et al*. [16]. Limited vertical connectivity was recently reported also in the Indo-Pacific coral *Seriatopora hystrix* [10], in the Caribbean coral *Montastraea cavernosa* [13] and in the candelabrum coral *Eunicea flexuosa* [12], stressing the role of depth and its related abiotic factors in determining genetic structure of the populations.

In the study area, a seasonal thermocline is located at about 40 meters depth [56,57]. Large fluctuations of the water temperature have been reported, changing from 21–23°C to 14–17°C in few hours [16,56]. In the same period, *E*. *singularis*, a gonochoric brooding species, releases mobile planulae larvae with a short pelagic larval duration [58,59]. The survival and dispersal potential of the planulae could be affected by temperature fluctuations, resulting in a reduced larval exchange between shallow and deep populations, that could lead to reproductive isolation (e.g. [11]). Nevertheless, as already observed by Padron [60] genetic structuring may be observed also when limited connectivity occurs. Moreover, limited habitat availability together with the patchy distribution of deep populations could promote isolation even if physical barriers to larval dispersal do not arise. Reproductive isolation along the depth gradient has been observed in other Mediterranean, Pacific and Caribbean corals (e.g. [6,10,12,13,61]. In the tropical coral *Seriatopora hystrix*, populations and associated symbiotic algae are highly structured across habitats on a single reef [10]. Endosymbiotic photosynthetic dinoflagellate were not found after visual inspection of histological sections of *E*. *singularis* colonies collected at 40–60 m depth [16]. The coral-symbiont interaction implies that assessment of larval dispersal and connectivity in corals need to be complemented by investigations on symbiont dispersal [62], because processes of symbiont transfer may impose limitations on the colonization and post settlement survival of coral offspring [10].

### Genetic connectivity along coast

No genetic structuring was observed among shallow populations of *E*. *singularis* along the coast of Cap de Creus [18]. Distance between the farthest populations analysed in this study is less than 15 km, this geographic scale may be to limited to detect genetic structure in this species, as found by Pey *et al*. [54], while comparing populations hundreds of km apart. Regional scale connectivity was also observed in *Eunicella verrucosa* [63]. Both *Eunicella* species are thought to have a limited dispersal, with larvae settling mainly close to the maternal colonies [59]. However, the widespread distribution of *E*. *singularis*, together with the observed high horizontal connectivity, suggests that marine coastal currents are likely to be the main vector for larval and or gamete dispersal in this species. In fact, in Cap de Creus the general circulation pattern is dominated by currents usually flowing from North to South, but intense and persistent Catalan eddies have also been registered in summer [57], when larvae are released [19].

### Preliminary phylogenetic analysis of *Eunicella*

Both microsatellites and ITS-1 showed a low level of the genetic variability. The observed number of alleles was in the same range as reported for *Eunicella singularis* [24,54] and *E*. *cavolinii* [55] populations. The rate of polymorphism and the success of microsatellite cross-species amplification has been related to the evolutionary divergence between species [64]. Quattrini et al. [65] suggest that microsatellite loci may resolve species boundaries of octocorals that have been separated by approximately 19 Ma (*Callogorgia delta* and *C*. *americana*). Within Mediterranean species of the genus *Eunicella* speciation events were not detectable by the available genetic markers and may be recent or still in progress. This former hypothesis is in agreement with the opening of the Strait of Gibraltar approx. 5.32 My, when marine conditions were restored by the massive flow of Atlantic water and species into the Mediterranean Sea [66].

Contrary to ITS2 [21], ITS1 seems more variable but still not enough to reveal a clear pattern of horizontal and vertical population structuring (see above). Moreover, the ITS1 was only able to discriminate between *E*. *racemosa* and the three Mediterranean *Eunicella* species, which clustered together in a single group. Similar results were obtained using mitochondrial markers MutS, COI and Igr1 segments, having the same sequence in *E*. *singularis*, *E*. *cavolinii* and *E*. *verrucosa*, while differing from *E*. *racemosa* (see S1 Material and Methods). *E*. *racemosa* is distributed along the Atlantic African coast from Angola to Morocco, it has very large colonies, up to 2 m high (López-González, pers. obs.). It is worth to notice that despite the large morphological differences between *E*. *racemosa* and the Mediterranean *Eunicella* species (see also [14]), mitochondrial markers (MutS, COI and Igr1) only differ in silent substitutions (not causing a change of aminoacid, see S1 Materials and Methods). Although, based on the current knowledge, a North Atlantic origin of the Mediterranean *Eunicella* species could be proposed, perhaps from a common ancestor close to *E*. *verrucosa* (the only *Eunicella* species in the boreal and in the lusitanic biogeographic regions). Further studies should include additional species from Ibero-Moroccan Gulf and the near Atlantic African coasts in order to get insights into the diversification of the *Eunicella* genus, and the origin of the Mediterranean endemism.

## Conclusions

Shallow benthic communities are commonly exposed to higher environmental stress compared to deeper ones, e.g. thermal stress events and storm-induced disturbance [67]. In tropical seas, it has been hypothesized that deep coral reefs can potentially act as (reproductive) refugia for several coral species [9], fostering the re-colonization of shallow areas after catastrophic events [68]. This hypothesis grounds on the existence of connectivity between shallow and deep sublittoral populations [9].

The present study showed genetic divergence between populations above and below about 40 m depth. As a consequence, the deep refugia hypothesis developed for the tropical seas seems to be not valid for Mediterranean gorgonians. In the Northwestern Mediterranean Sea, extreme thermal stress in shallow waters has recently resulted in mass mortalities of benthic invertebrate species, including gorgonians (e.g. [69]). However, re-colonization and population’s recovery after a mass mortality event cannot be supported by larvae from deeper colonies, but can rely only on larvae produced by nearby shallow water populations. Concerning the taxonomy of the genus *Eunicella* in the Mediterranean Sea, low levels of mitochondrial genome evolution [20], and low polymorphism of nuclear markers, do not allow to validate the species status neither of the two morphotypes, nor of the three *Eunicella* species. The large morphological variability of *Eunicella* genus, as in other Octocorallia genera, leads uncertainty regarding species boundaries and classification. Methods based on next-generation sequencing (e.g. RAD sequencing, [70]) may provide further insights into the evolutionary relationships in recalcitrant taxa such as *Eunicella*.

## Supporting Information

S1 Dataset Microsatellite data set. The allele sizes in 6 loci studied in 13 *E*. *singularis* populations.
(XLS)

S1 Materials and Methods Materials and Methods and Results. DNA extraction of the *Eunicella* species and PCR amplification of the three mitochondrial markers and their genetic variability.
(DOCX)

S1 Table Pairwise genetic p distance among *Eunicella* species. Pairwise genetic p distance (Dp) among *Eunicella* species including the sampled populations of *Eunicella singularis*.
(DOCX)
