# Supplementary material for: Limited Genetic Connectivity between Gorgonian Morphotypes along a Depth Gradient
Source: PLoS One. 2016 Aug 4;11(8):e0160678. doi: 10.1371/journal.pone.0160678 (PMC4973999; doi:10.1371/journal.pone.0160678)
Supplement: S1 Materials and Methods — DNA extraction of the Eunicella species and PCR amplification of the three mitochondrial markers and their genetic variability. (DOCX) [file pone.0160678.s002.docx]

**S1** **Materials and Methods.** DNA extraction and PCR amplification of the three mitochondrial markers and their genetic variability.

**Materials and Methods**

Small fragments of one colony of *E. singularis* from CCR3_20, CCR3_40; CCR3_50; CCR3_60; of *E. cavolinii*, of *E. verrucosa* and *E. racemosa* of the same sampling sites as in the Manuscript were also used to evaluate their variability in three mitochondrial region: mtMutS (msh1), Igr1 and COI.

Total genomic DNA was extracted from the ethanol-preserved fragments using the E.Z.N.A. DNA kit (OmegaBiotech) following the manufacturer’s instructions. The mtMutS (msh1), and Igr1+COI mitochondrial regions were sequenced as an octocoral barcode proposed by McFadden et al. (2011) for comparative purposes. The start of the mtMutS (msh1) region was amplified using the primers ND42599F and MUT3458R (France and Hoover 2002; Sánchez et al. 2003). *Igr1*+*COI* region was amplified using the primers COII8068F and COIOCTR (McFadden et al. 2004; France and Hoover 2002). PCR profiles, purification protocol and sequencing as described in López-González et al. (2015).

Sequence genetic diversity was estimated as number of haplotypes (h), haplotype and nucleotide diversity (Hd and π, respectively). All these parameters were calculated through DnaSP v5 (Librado & Rozas 2009). Distance matrixes of sequence divergence among the species were calculated as p distance (Dp) in MEGA v.5.05 (Tamura et al. 2011).

**Results**

The length of the amplified mitochondrial regions was 786 pb for COI, 737 pb for msh1 and 112 pb for Igr1. For each of the three regions only two haplotypes were detected: one common to *E. singularis* individuals, *E. cavolinii* and *E. verrucosa* and the other private for *E. racemosa*. The two COI haplotypes differ for 4 silent nucleotide substitutions (Dp *_E. racemosa_* _vs._ *_E. cavolinii, E.singularis, E. verrucosa_* = 0.00509); the msh1 haplotypes for one silent substitution (Dp *_E. racemosa_* _vs._ *_E. cavolinii, E.singularis, E. verrucosa_* = 0.00136) and the Igr1 haplotypes diverge for 6 silent nucleotide substitutions (Dp *_E. racemosa_* _vs._ *_E. cavolinii, E.singularis, E. verrucosa_* = 0.05357).

All the sequences were deposited in GenBank with accession numbers from KX051564 to KX051577.

**References**

France S, Hoover L. DNA sequences of the mitochondrial COI gene have low levels of divergence among deep-sea octocorals (Cnidaria: Anthozoa). Hydrobiol. 2002; 471:149-155

Librado P, Rozas J. DnaSP v5: a software for comprehensive analysis of DNA polymorphism data. Bioinformatics. 2009; 25:1451–2.

López-González PJ, Grinyó J, Gili J-M. *Chironephthya mediterranea* n. sp. (Octocorallia, Alcyonacea, Nidaliidae), the first species of the genus discovered in the Mediterranean Sea. Mar Biodiv. 2015; 45:667–688. DOI 10.1007/s12526-014-0269-5

McFadden CS, Tullis ID, Hutchinson MB, Winner K, Sohm JA. Variation in coding (NADH dehydrogenase subunits 2, 3, and 6) and noncoding intergenic spacer regions of the mitochondrial genome in Octocorallia (Cnidaria: Anthozoa). Mar Biotechnol. 2004; 6:516-526

McFadden CS, Benayahu Y, Pante E, Thoma JN, Nevarez PA, France SC. Limitations of mitochondrial gene barcoding in the cnidarian sub-class Octocorallia. Mol Ecol Resour. 2011; 11:19-31

Sánchez J, McFadden CS, France S, Lasker H. Molecular phylogenetic analyses of shallow-water Caribbean octocorals. Mar Biol. 2003; 142:975-987

Tamura K, Peterson D, Peterson N, Stecher G, Nei M, Kumar S (2011) MEGA5: molecular evolutionary genetics analysis using maximum likelihood, evolutionary distance, and maximum parsimony methods. Mol Biol Evol. 2011; 28:2731–273
