# Supplementary material for: Limited Genetic Connectivity between Gorgonian Morphotypes along a Depth Gradient
Source: PLoS One. 2016 Aug 4;11(8):e0160678. doi: 10.1371/journal.pone.0160678 (PMC4973999; doi:10.1371/journal.pone.0160678)
Supplement: S1 Table — Pairwise genetic p distance (Dp) among Eunicella species including the sampled populations of Eunicella singularis. (DOCX) [file pone.0160678.s003.docx]

**S1 Table.** Pairwise genetic p distance (Dp) among *Eunicella* species including the sampled populations of *Eunicella singularis*
